# Reduction of carbonic anhydrase activity is associated with amelioration of obstructive sleep apnea

**DOI:** 10.1007/s11325-025-03430-z

**Published:** 2025-09-04

**Authors:** Ali M. Komai, Saliha Musovic, Kaj Stenlöf, Ludger Grote, Ding Zou, Jan Hedner

**Affiliations:** 1 Center for Sleep and Wake Disorders, Sahlgrenska Academy, Gothenburg University, Gothenburg, Sweden; 2 Department Internal Medicine, Carlanderska Hospital, Gothenburg, Sweden; 3 Sleep Disorders Center, Respiratory Medicine, Sahlgrenska University Hospital, Gothenburg, Sweden; 4 Center for Sleep and Vigilance Disorders Institute of Medicine, University of Gothenburg, Medicinaregatan 8B, Box 421, Gothenburg, SE-40530 Sweden

**Keywords:** Acetazolamide, Bicarbonate, Cerebrospinal fluid, Diagnosis, Sleep disordered breathing, Sulthiame, Therapy

## Abstract

**Background:**

The carbonic anhydrase (CA) enzyme plays an important role in the equilibration of carbon dioxide and bicarbonate (HCO_3_) under the production of H^+^ ions. Emerging evidence suggest that CA activity may play a fundamental regulatory role on respiratory control mechanisms in obstructive sleep apnea (OSA). Clinical trials suggest that CA inhibitors significantly reduce OSA.

**Methods:**

Data from three separate cohorts of healthy volunteers and patients with OSA were used to quantify CA activity in whole blood and cerebrospinal fluid (CSF). The influence of the CA inhibitory drugs acetazolamide and sulthiame, on CA activity in-vitro/in-vivo, was assessed. The association between CA-inhibitor plasma concentration and HCO_3_, as well as the influence of HCO_3_ on the apnea-hypopnea severity was determined.

**Results:**

Stability of CA activity in stored blood was high. CA activity in whole blood contained five times higher activity compared with CSF. The CA-inhibitory drugs dose-dependently reduced CA activity in-vitro/in-vivo. The CA inhibitor sulthiame reduced venous HCO_3_ concentration (*P* = 0.022). The reduction of HCO_3_ was linked to improvement of OSA (*P* = 0.040).

**Conclusions:**

CA-inhibitory drugs reduced CA activity in whole blood suggesting a therapeutic role of CA inhibition in OSA. The findings also suggest that an activated CA system may constitute a pathophysiological mechanism in some forms of OSA.

**Clinical trial registration:**

N/A.

**Supplementary Information:**

The online version contains supplementary material available at 10.1007/s11325-025-03430-z.

## Introduction

Obstructive sleep apnea (OSA) is a clinical disorder associated with comorbidity including daytime sleepiness, cognitive disorder as well as cardiovascular and metabolic disease. The carbonic anhydrase (CA) enzyme plays an important role for equilibration of carbon dioxide and bicarbonate (HCO_3_) under the production of H^+^ ions [1]. Recent data suggests that the CA enzyme may play an important regulatory role in OSA by modifications of respiratory control mechanisms [2, 3]. 

CA is widely spread in the body and catalyzes a fundamental chemical reactions associated with vital processes in man [4]. CA occurs in at least 16 isoforms which adds to the diversity of this regulatory system [5]. At least three CA isoenzymes, CA-II, CA-IX, and CA-XII are thought to participate in pH regulation as well as various metabolic processes [6]. The regulation of pH, including provision of bicarbonate or H^+^ ions for electrolyte secretion, as well as specific metabolic pathways such as in lipogenesis, gluconeogenesis and ureagenesis provides a plethora of mechanisms that have triggered a development of CA inhibitory drugs [5]. The clinical indications include diuretic therapy, glaucoma, high altitude disease, and epilepsy. However, CA mechanisms have also been explored in the management of cancer [7], neuropathic pain [8], sleep apnea [9, 10], migraine [11], elevated intracranial pressure [12], and cerebral ischemia [13].

The current study aimed to explore the usefulness of a conventional analysis tool for CA activity in biological fluids, in particular venous hemolyzed blood. We hypothesized that a modified CA activity in the control of breathing might play a causal role for the occurrence or maintenance of the disorder. We investigated the accuracy and stability of blood samples and determined CA activity in serum, whole blood hemolysates and in cerebrospinal fluid (CSF). To address the clinical relevance of CA activity in OSA, we investigated the impact of sulthiame (STM), a CA inhibitory drug under development in OSA, on HCO_3_ as a surrogate measure of CA activity in sleep disordered breathing [14, 15].

## Methods

### Subjects and biological samples

#### Data from three separate cohorts were analyzed

A. In the first protocol, we collected blood samples for analysis from the antecubital vein in four healthy, male, normal weight subjects after a short rest in the supine position Samples were collected in EDTA or heparinized tubes. While some tubes were immediately cold centrifuged at 2000 rpm (ALC4237R) to generate cold-stored plasma, others were left to sediment at room temperature for the stability assessments. Samples were pipetted into aliquots of 1 ml and immediately frozen at -70 °C to constitute whole blood hemolysates in the subsequent experiments.
B. Blood samples from four control subjects and eight overweight patients (cohort 2) undergoing a CSF tap were obtained. The study setting was a previous clinical trial of weight loss therapy performed at the Department of Endocrinology, Sahlgrenska University hospital. Inclusion criteria were male sex, age above 18 years, a body mass index (BMI) between 27.5 and 37.0 kg/m^2^, and weight stability. Significant cardiometabolic disease constituted an exclusion criterion. CSF sampling was performed in the morning (at about 06.00) according to standardized procedures with the examined subject in a lateral recumbent position and lumbar puncture at the L3–L4 or L4–L5 interspaces with a standard needle (Sprotte standard needle with introducer, 0.7 mm, 22-gauge, 90/120 mm; Rusch Inc., Duluth, GA). Blood samples were taken shortly after the CSF sampling at about 08.00 h. The subjects were fasting overnight. The CSF and serum samples were immediately placed on ice and centrifuged at 4 °C. Samples were frozen in aliquots at -70 °C.
C. The third cohort was a randomized, placebo controlled clinical phase IIA trial investigating the effect of STM 200 mg, 400 mg or corresponding placebo on sleep disordered breathing in patients with moderate to severe OSA. A detailed description of the experimental procedures in this trial has been published elsewhere [9]. In this trial drug dosing was titrated during a two plus two-week period. Samples were obtained from a total of 59 patients adhering to the study protocol at baseline and follow-up. A total of nine patients in the highest dose group left the study prematurely. The safety and efficacy blood sampling in this study was extensive and included, among other clinical chemistry assays also serum HCO_3_, capillary blood gases, chloride concentration and the calculated anion gap without adjustment for potassium concentration.

Subjects in the three groups gave their written informed consent for the study and the ensuing analysis. The analyzed materials were collected during 2022, 2021 and 1997-98, respectively, and the protocols used for the completion of the current approvals had been reviewed by the local independent Institutional review board (R409-97 and R268-98, Dnr 2019–05096 and Dnr 045–18) and conducted in accordance with the declaration of Helsinki and local regulations.

### CA activity assessment using a commercially available assay kit

We followed the protocol specified in the commercially available assay kit (BioVision, Catalog # K472-100). (see detail in e-supplement)

#### Experiment 1: stability of the standard curve

To remove the background absorbance provided by the reagent diluent, we used a blank sample. A positive control provided in the kit (according to the manufacturers recommendation) was used in each measurement to guarantee the validity of the obtained data. A 6-point standard curve was prepared from a 2mM nitrophenol stock solution, provided by the assay kit, to generate the following concentrations: 0, 8, 16, 24, 32 and 40 nmol/well.

#### Experiment 2: analysis of dilution factor in blood samples

A dilution series was conducted, on hemolyzed whole blood samples from two subjects. We determined the proper necessary dilution to ensure detectable absorbance values (OD) within the nitrophenol range of the standard curved (0–40 nmol). Samples were diluted 1000, 10 000 or 15 000 times.

#### Experiment 3: analysis two different hemolysis protocols during CA inhibition

In this experiment blood from the same subject was analyzed under otherwise identical conditions, but different hemolysate preparation protocols, A and B, were used. In protocol A, blood samples were collected in heparinized tubes and then immediately frozen at -80 °C. Samples were thawed followed by three pipetting/vortexing rounds prior to the analysis of CA activity. In protocol B, the manufacturer’s protocol was used. One volume of the RBC fraction was washed with two columns of ice-cold saline solution (1mM Tris, pH 8.0, 200 mM NaCl) and the collected sample was centrifuged once more at 3000 x g for an additional 5 min at 4 °C. The RBC fraction was washed in three volumes of ice-cold buffer (1 mM Tris, pH 8.0) and placed on ice for 10 min. Complete lysis was achieved by placing the samples in -80 °C for 15 min. The lysis suspension was centrifuged one last time at 15 000 x g for 15 min to remove cellular debris. The supernatant was collected for immediate assay.

#### Experiment 4: stability of the CA sample activity in room air and ambient temperature

We determined the stability of CA activity in blood samples collected and stored at room temperature but frozen at different time points after collection: Samples were centrifuged and aliquoted immediately, 30 min, and 4 h after sampling at room air and stable ambient temperature. The samples were thereafter frozen at -80 °C. After thawing CA activity was analyzed according to protocol A for two different subjects (S1 and S2).

#### Experiment 5: inhibition of CA activity in-vivo by STM

CA activity was measured in blood collected between 9 and 11 a.m. at the baseline day as well as at days 1, 2 and 3 with the CA-inhibitor STM in four healthy subjects. Samples were obtained with the subjects in a semi-reclined body position. All subjects were free of medication and had no other medical history of significance to this protocol. The daily dose of STM (100 mg tablets) was increased stepwise from 100 mg to 200 mg and 300 mg. CA activity was assessed in hemolysed whole blood in accordance with the procedure described above. CA activity levels were adjusted with total protein content using Bradford assay [16, 17].

#### Experiment 6: analysis of CA in blood and cerebrospinal fluid

CA activity was analyzed in CSF and whole blood samples, collected and prepared according to protocol A, under standardized procedures in a matched fashion (same subjects) at approximately 06:00 and 08:00 a.m., respectively. The samples analyzed in experiment five were originally obtained from a group (*n* = 8) of otherwise healthy patients with moderate to severe obesity consenting to the sampling and analysis protocol. Samples analyzed in the current study were collected before any introduction of study drug.

#### Experiment 7: analysis of STM and bicarbonate in OSA patients receiving 200 or 400 mg STM

In a phase II clinical trial [9], blood samples were collected immediately prior to the last dosing which occurred within 60 min prior to bedtime to reflect the lowest drug concentration value at steady state. Both STM activity and HCO_3_ concentration (mmol/l) were analyzed at baseline and at the end of the medication period. The bicarbonate concentration was determined in accordance with the standard procedure at the accredited central laboratory of the Sahlgrenska University Hospital. The STM concentration was determined by a liquid chromatography technique with tandem mass spectrometry (LC-MS/MS) at the Analytisches Zentrum Bipharm (Berlin, Germany) [9]. Samples from patients with OSA (*n* = 27) were collected in the evening prior to a sleep study. Following centrifuging and pipetting they were frozen at -70 °C.

### Sleep studies

In this trial [9], sleep studies were performed following a standardized in-lab polysomnography routine (Embla A10 system, Medcare Flaga, Iceland) and manually scored according to the AASM criteria [18]. Hypopneas reaching a threshold of 4% oxygen desaturation were used to quantify define hypopnea and the severity of the OSA condition.

### Statistical methods

Statistical analysis was performed using SPSS version 28 (IBM, Armonk, NY, USA). Data are presented as mean ± SEM. Pearson and Spearman rank correlation analysis were performed to analyze the potential association between CA activity in blood and CSF, STM concentrations, bicarbonate and OSA severity.

## Results

### Experiment 1: stability of the standard curve and dilution steps

**Fig. 1:**
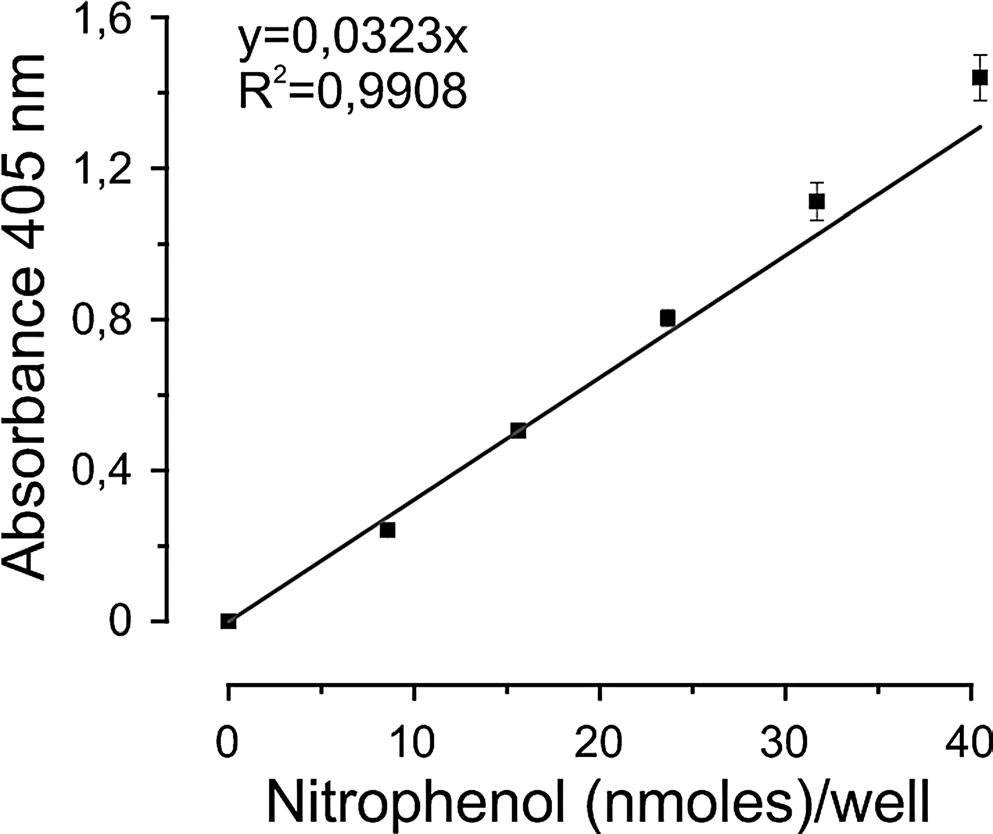
Data from nine separate independent experiments to determine the stability and integrity of the CA activity assay. Three separate assay kits were evaluated for six dose levels of nitrophenol. A low variance was obtained for the absorbance at 405 nm (y-axis) and there was no difference in absorbance at the various concentration steps between individual experiments

Nine separate independent experiments were performed to determine the stability and integrity of the CA activity assay. A low variance was obtained for the absorbance (OD) values at the separate points of the nitrophenol standard curve (Fig. 1) and there was no difference in absorbance at the various concentration steps between the individual experiments (data not shown). Three separate assay kits with individual lot numbers were evaluated for the six dose levels of nitrophenol and there was only minor inter-experiment variability (Coefficient of variability (CV < 10%) between experiments at the higher concentrations of nitrophenol.

### Experiment 2: analysis of dilution factor in blood samples

**Fig. 2:**
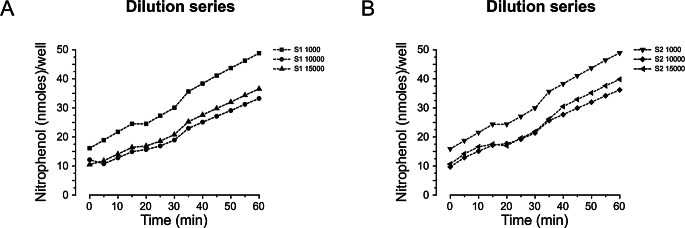
Nitrophenol concentration (nm) resulting from CA esterase activity measured with 5 min intervals for a total of 60 min. Hemolysates were collected from two subjects (S1, S2) and samples were diluted 1000, 10 000 and 15 000. Note that CA activity showed a linear activity over time and within the assay concentration limit when diluted to 1:10000 or 1:15000. A dilution of at least 10 000-fold appeared to be appropriate

A dilution of 1:1000 reached the assay concentration limit independent of the hemolysate protocol S1 or S2 (Fig. 2). When the whole blood sample was diluted to diluted to 1:10000 or 1:15000-fold, CA activity showed a linear activity over time within the assay concentration limit. A dilution of at least 10 000-fold appeared to be appropriate for this kit.

### Experiment 3: analysis of two different hemolysis protocols

**Fig. 3:**
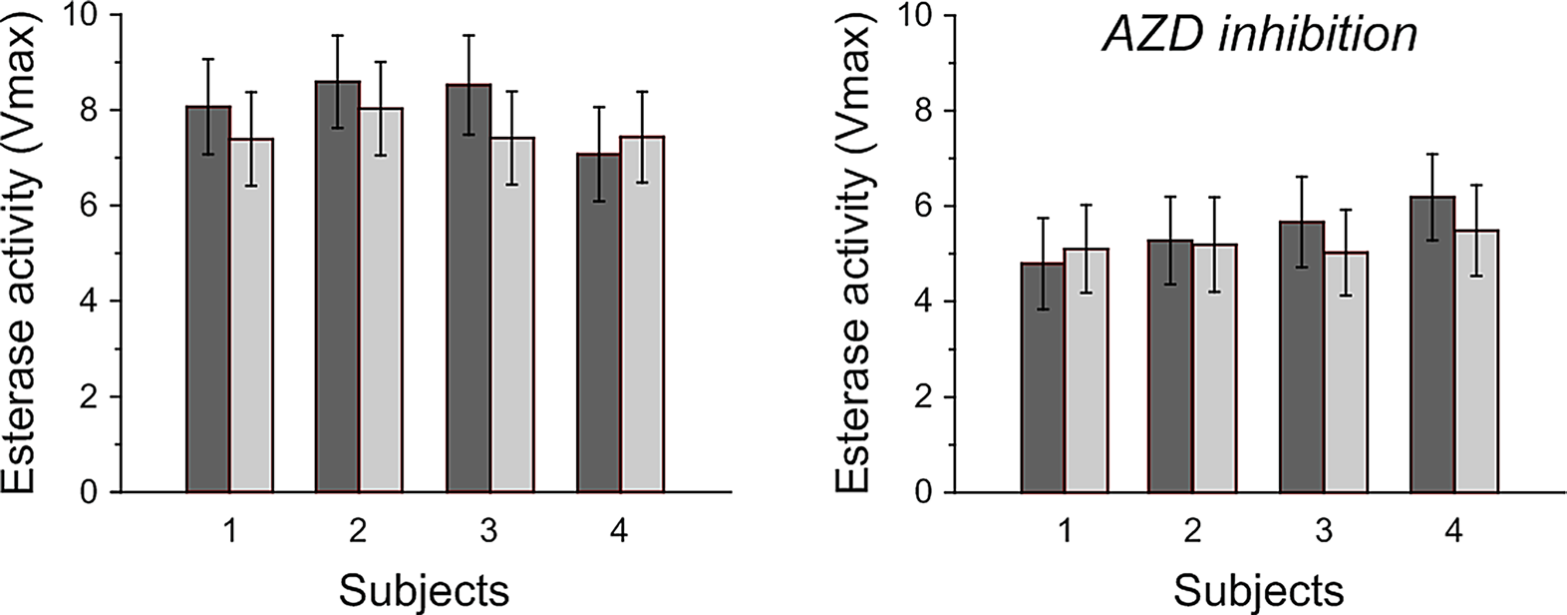
CA activity in hemolysed venous blood following hemolysis protocol **A** (dark grey) and **B** (light grey) at baseline (left panel) and after addition of the CA activity inhibitory drug acetazolamide (AZD, right panel)

Total esterase activity was substantially reduced in all four subjects when AZD was added to the blood sample (Fig. 3). Data confirmed the similarity of the two hemolysis protocols also in the presence of the CA inhibitor AZD. There was no systematic difference between the hemolysate protocols A and B.

### Experiment 4: time window from blood sampling to freezing

**Fig. 4:**
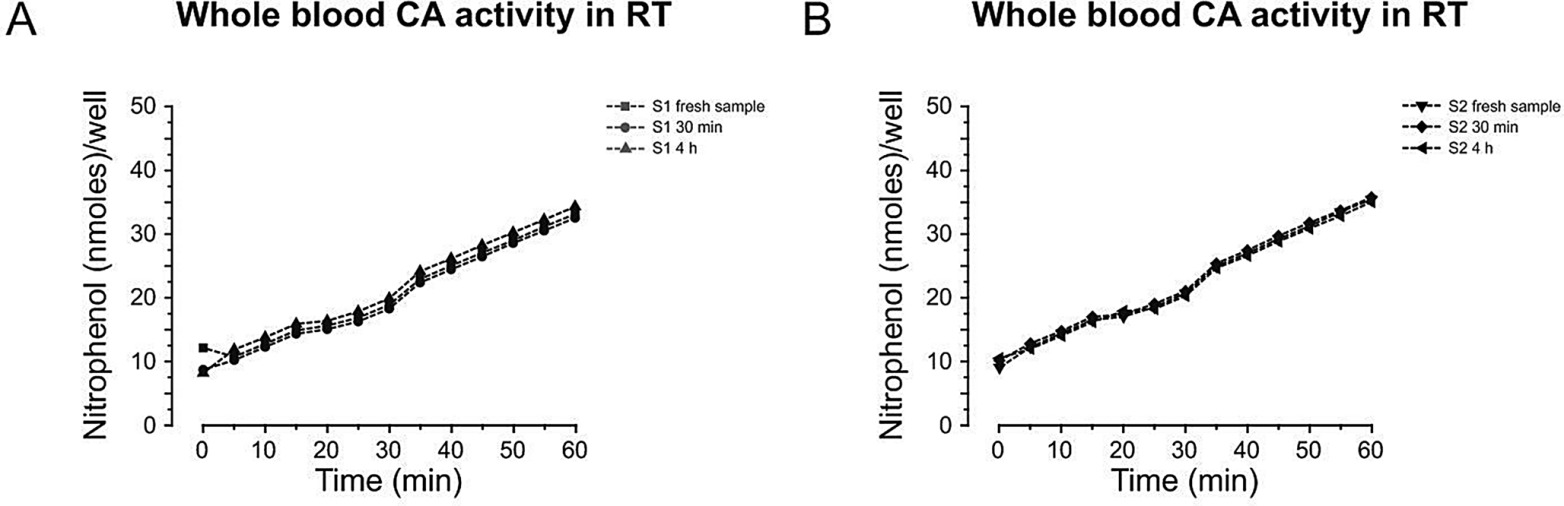
Stability of CA activity was determined in samples frozen immediately, 30 min–4 h after collection. The blood samples were stored at room temperature before freezing. Results are shown for **A**) subject 1 (S1) and **B**) subject 2 (S2)

Stability of CA activity was determined in blood samples immediately upon collection or following collection and storage at room temperature for 30 min–4 h before freezing (Fig. 4A and B). CA activity was not compromised by extended storage at room temperature and found to be stable across the 4-hour observation period.

The CV values are comparable when CA activity was measured in blood collected in either heparin or EDTA tubes (samples from the same subjects) and the variance in absorbance obtained with two different spectrophotometers at repeated measurements was less than 10%. To investigate the interplate (experimental) variability, the same blood samples were used for experiments at separate occasions. Repeated measurements of CA activity were feasible and resulted in values with low variance (data not shown).

### Experiment 5: inhibition of CA activity in-vivo by STM

**Fig. 5:**
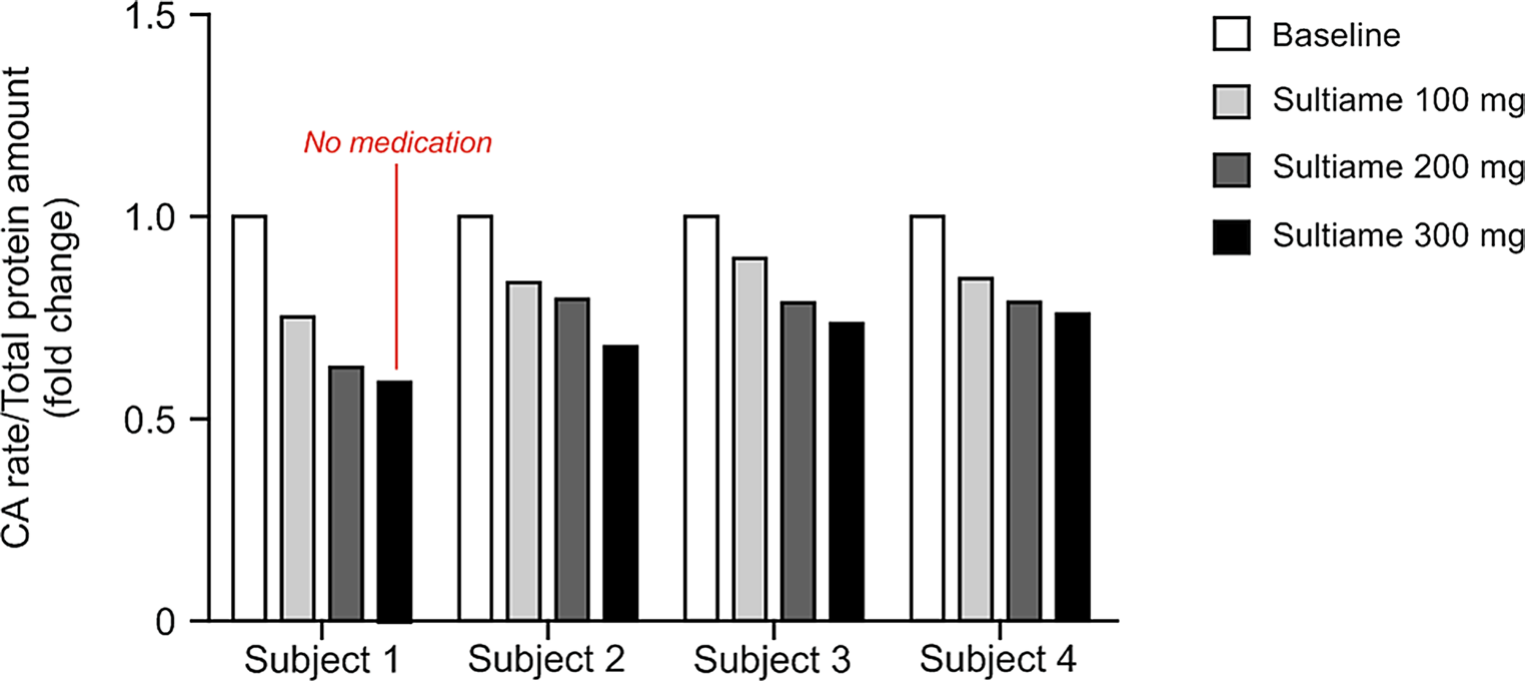
Venous CA activity in four healthy subjects receiving the CA inhibitor STM in escalating doses from 100 to 300 mg daily for three days. Note deviating pattern in subject one. Blood sampling was performed immediately prior to scheduled tablet intake

Blood samples for CA activity assessment were collected daily prior to intake of STM. STM was well tolerated in three of the subjects while subject 1 developed nausea after 200 mg of STM. The 300 mg dose was therefore withheld (but a blood sample was obtained on day three) for this subject. CA activity in hemolyzed blood was reduced by 25–35% in a dose dependent fashion after STM. (Fig. 5). The pattern of reduction was similar in all four tested subjects and the suppression of STM activity was maintained for at least 48 h considering the reduced activity recorded in subject one on day three.

### Experiment 6: analysis of CA activity in hemolyzed blood and CSF

**Fig. 6:**
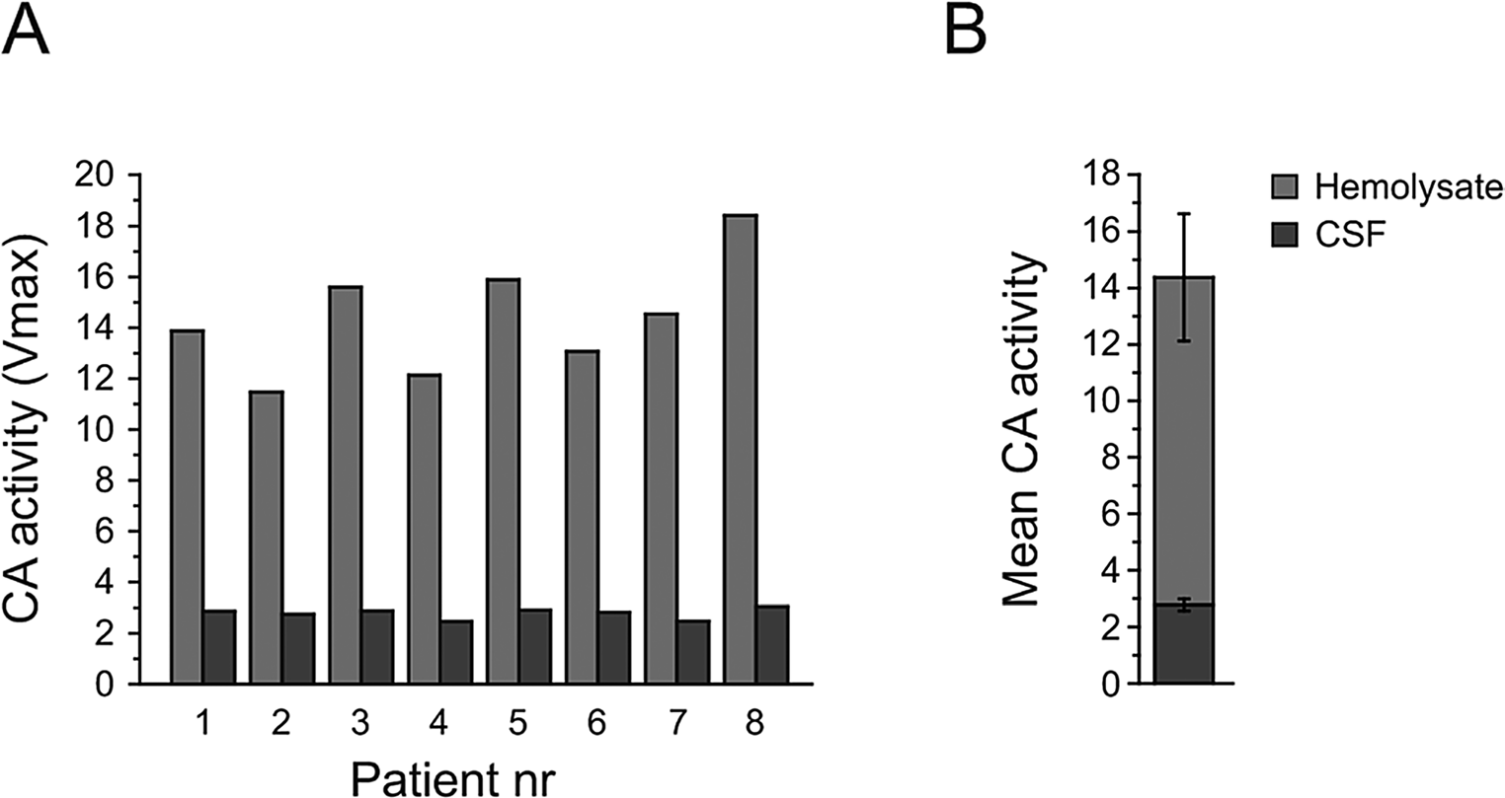
CA activity in CSF (dark grey) and whole blood (light grey) in eight moderately obese males (individual data shown in **A**), pooled data shown in **B**). CSF sampled in the morning (approximately 06.00) according to standardized procedures with the subject in a lateral recumbent position

CA activity was present in all CSF samples and the mean quantified CA activity in whole blood was 14.38 ± 2.25 compared with 2.78 ± 0.21 in CSF and there was a clear correlation between blood and CSF CA activity (Spearman, r_s_ = 0.81, *P* = 0.015). Approximately 20% CSF CA activity in proportion to matching hemolysed whole blood activity was remarkably similar in all tested subjects. The whole blood CA activity appeared to differ between subjects to a slightly higher extent. **(**Fig. 6**)**.

### Experiment 7: analysis of STM and HCO_3_ serum concentration in OSA patients receiving 200 or 400 mg STM

**Fig. 7:**
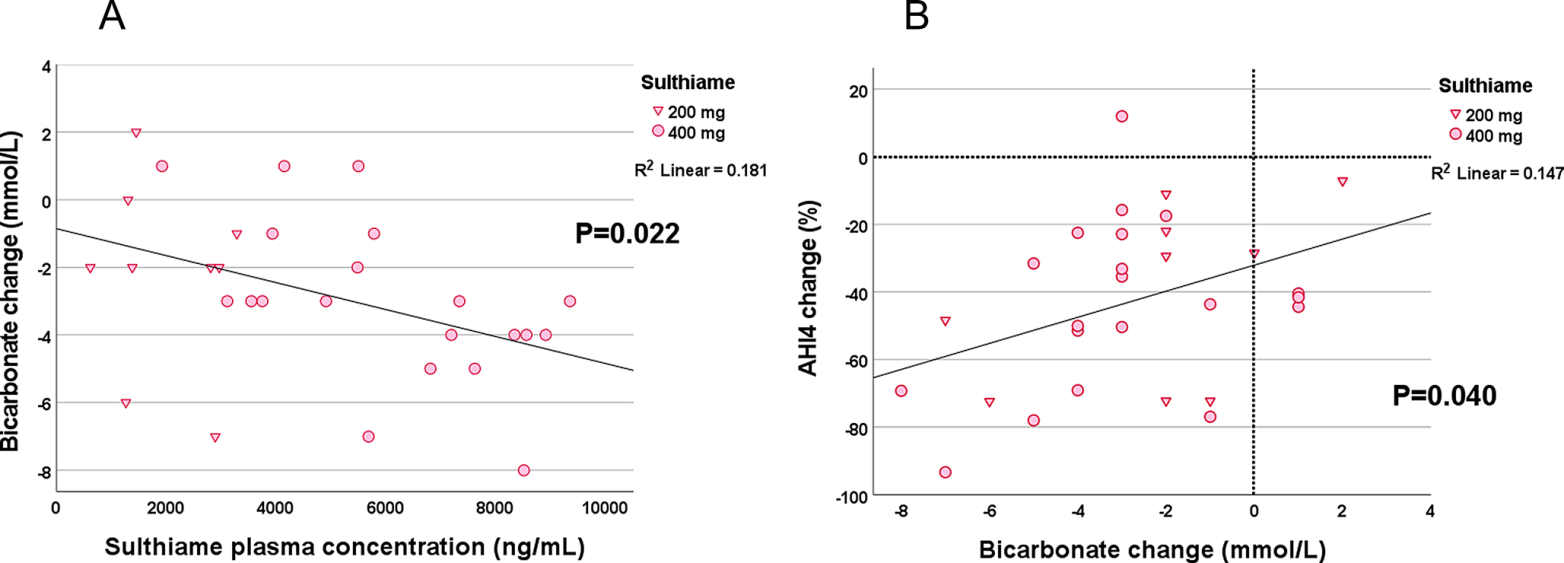
**a** (upper) and **b** (lower). Associations between STM plasma concentration (ng/ml) and bicarbonate change (mmol/l) as well as change in bicarbonate concentration and percent change of AHI4 following the 200 mg (triangles) or 400 mg (squares) dosage regimen. Note the aggregation of values in the lower left quadrant suggesting a consistent reduction of bicarbonate following sulthiame

As expected, there was a linear reduction in bicarbonate concentration along with increasing STM plasma concentration (Pearson, *P* = 0.022) (Fig. 7a). A reduction of HCO_3_ was also associated with the change in the apnea-hypopnea index (4%, AHI4) (Pearson, *P* = 0.040) (Fig. 7b). In fact, 24/29 (83%) of the patients receiving STM in this study reduced both HCO_3_ concentration and the AHI4 suggesting a tight association between the two.

## Discussion

Our study provides several important findings. First, CA activity can be accurately quantified in whole blood and CSF in healthy subjects and in patients with OSA. Samples were stable in-vitro for up to 4 h at room temperature and there was a 5-fold difference in CA activity in whole blood and CSF upon parallel sampling in otherwise healthy obese subjects. Second, modification of enzyme activity by a CA inhibitor STM dose dependently reduced whole blood CA activity in healthy controls. Third, when clinically applied in patients with OSA, STM modified the CA system and reduced the breathing disorder.

CA is widely distributed in the human body including the central nervous system, for example in oligodendrocytes as well as in the chemoreceptive areas of the medulla oblongata [19]. Other, peripheral tissues exhibiting CA activity include those directly or indirectly involved in the regulation of ventilation and acid-base balance such as erythrocytes, carotid bodies, lung capillary endothelium and renal tubular cells [4]. Indeed, the wide distribution of CA expression suggests a plethora of biological effects of drugs interacting with CA inhibitory properties [5]. Several studies have analyzed CA activity in plasma or whole blood using different methodologies but there are, to the best of our knowledge, no previous studies addressing individual, simultaneous activity in whole blood and CSF or changes in activity following controlled, incremental dosages of drugs with CA inhibitory properties.

Our paper describes a method to accurately monitor CA activity with particular emphasis on hemolysed whole blood. This analysis suggested a considerable CA activity in whole blood that mainly may be ascribed to CA isoenzyme II, an enzyme demonstrated in human erythrocytes [20]. We did not assess specific CA isoenzymes in this study but a similar order, albeit lower, CA activity was found in CSF. These two compartments may be particularly important in the current context as they reflect mechanisms fundamental for the control of respiration/ventilation at sea level and high altitude, two circumstances known be influenced by drugs with CA inhibitory properties [9, 21, 22]. 

Indeed, a recent clinical trial of STM in patients with OSA demonstrated a considerable improvement of breathing during sleep and a simultaneous reduction of blood CA activity [9, 23]. Although we could not confirm this association at the individual patient level, there was a pronounced reduction of the apnea-hypopnea Index (AHI) in all four investigated plasma STM concentration quartiles [3, 23]. In the current study, we report a significant association between the HCO_3_ reduction and the AHI improvement in STM treated subjects. The findings support the notion of developing a novel principle for drug development in OSA. However, the exact pharmacological mechanism(s) behind the beneficial effect in OSA remains to be better understood.

The biological relevance of CA isoenzymes in the central nervous system may be illustrated by the potent CA inhibitory properties that characterize several antiepileptic drugs. These CA inhibitors promote a process of acidification via mechanisms in the central nervous system related to the activity the CA isoforms II, IV, V, VII and XII. Important isoenzymes in peripheral tissue, for example II, VII, IX and XII, have been identified in erythrocytes, skeletal muscle, gastrointestinal mucosa and kidney, respectively [4]. Dominant effects in the periphery, also demonstrated in our study, include a facilitation of renal HCO_3_ secretion which operates in the direction of a metabolic acidosis and an altered CO_2_ reserve. It cannot be excluded that respiratory control mechanisms are fundamentally altered to play an important pathogenetic role in individuals with certain forms of sleep disordered breathing. Further studies on the exact role of CA in the respiratory control system as well as how its impact on OSA are warranted.

Recent research in OSA has led to several more or less well defined endotypic traits in OSA [24]. Various analysis tools may typically separate characteristics such as loop gain, arousal threshold and ventilatory response to arousal as well as various measures of upper airway collapsibility [25]. A recent study form our laboratory suggested that STM mainly reduced the ventilatory instability (loop gain) and, somewhat unexpectedly, improved the upper airway collapsibility [3]. A better understanding of the characteristics of such endotypic traits might reveal a selective effect of CA inhibition in subgroups of OSA. However, there is a relative lack of studies investigating how such endotypic characteristics of the upper airway may be affected by CA therapy in OSA. Future studies of these associations might facilitate the introduction of precision medicine in the treatment of OSA [26]. 

Our study has several strengths as well as weaknesses. First, we demonstrated a specific analytical method rather than comparing several analysis techniques. An important technical detail is that hemoglobin concentration was corrected for in the current study. Both in-vitro and in-vivo, experiments supported a significant influence of CA inhibitors in sleep disordered breathing. The study group in the clinical evaluation was small but on the other hand the between subject variation of CA activity was low. Second, data from both healthy volunteers and patients with OSA was included. The use of CSF and blood samples as well as studies on two separate CA inhibitors further increased the robustness of the findings. Future studies are needed to investigate if various pathological conditions, such as OSA, might be associated with a primary modification of CA activity [2]. The whole blood CA activity was assessed in the study. Additional studies in this field are warranted to better understand the relative contribution of various CA isoenzymes in whole blood and other tissue. For instance, a recent study found that a deviating concentration of the isoenzyme CA-IX may provide a future target in terms of a biomarker to identify severe OSA or patients particularly suitable for a drug with CA inhibitory properties [27]. Interestingly, there was a strong correlation between hypoxemia (mean SaO_2_) and CA-IX concentration in that study. Such insights may be useful to investigate bioequivalence between various drugs that may be developed in this context.

It is concluded that there is considerable CA activity in hemolyzed whole blood. Activity was modified by drugs with CA inhibitory properties. This method may be useful to monitor CA activity in various clinical conditions characterized by altered acid-base mechanisms and differences in activity suggest that CA related mechanisms may be a useful target for drug development in OSA [28]. 

## Supplementary Information

Below is the link to the electronic supplementary material.

Supplementary Material 1

## Data Availability

The datasets analyzed during the current study are available from the corresponding author upon reasonable request.

## Declarations

### Permission to reproduce material from other sources

N/A.

## References

[1] Maren TH (1967) Carbonic anhydrase: chemistry, physiology, and Inhibition. Physiol Rev 47(4):595–781 4964060 10.1152/physrev.1967.47.4.595

[2] Wang T, Eskandari D, Zou D, Grote L, Hedner J (2015) Increased carbonic anhydrase activity is associated with sleep apnea severity and related hypoxemia. Sleep 38(7):1067–1073 25845687 10.5665/sleep.4814 PMC4481006

[3] Hoff E, Strassberger C, Zou D, Grote L, Stenlof K, Hedner J (2024) Modification of endotypic traits in OSA by the carbonic anhydrase inhibitor sulthiame. Chest 165(3):704–715 37776971 10.1016/j.chest.2023.09.022

[4] Supuran CT (2008) Carbonic anhydrases–an overview. Curr Pharm Des 14(7):603–614 18336305 10.2174/138161208783877884

[5] Mishra CB, Tiwari M, Supuran CT (2020) Progress in the development of human carbonic anhydrase inhibitors and their Pharmacological applications: where are we today? Med Res Rev 40(6):2485–2565 32691504 10.1002/med.21713

[6] Supuran CT (2018) Carbonic anhydrase inhibitors as emerging agents for the treatment and imaging of hypoxic tumors. Expert Opin Investig Drugs 27(12):963–970 30426805 10.1080/13543784.2018.1548608

[7] McDonald PC, Chia S, Bedard PL, Chu Q, Lyle M, Tang L et al (2020) A phase 1 study of SLC-0111, a novel inhibitor of carbonic anhydrase IX, in patients with advanced solid tumors. Am J Clin Oncol 43(7):484–490 32251122 10.1097/COC.0000000000000691 PMC7323835

[8] Supuran CT (2016) Carbonic anhydrase Inhibition and the management of neuropathic pain. Expert Rev Neurother 16(8):961–968 27211329 10.1080/14737175.2016.1193009

[9] Hedner J, Stenlof K, Zou D, Hoff E, Hansen C, Kuhn K et al (2022) A randomized controlled clinical trial exploring safety and tolerability of sulthiame in sleep apnea. Am J Respir Crit Care Med 205(12):1461–1469 35202553 10.1164/rccm.202109-2043OC

[10] Ni YN, Holzer RC, Thomas RJ (2023) Acute and long-term effects of Acetazolamide in presumed high loop gain sleep apnea. Sleep Med 107:137–148 37178545 10.1016/j.sleep.2023.04.010

[11] Mohammadianinejad SE, Abbasi V, Sajedi SA, Majdinasab N, Abdollahi F, Hajmanouchehri R et al (2011) Zonisamide versus topiramate in migraine prophylaxis: a double-blind randomized clinical trial. Clin Neuropharmacol 34(4):174–177 21738025 10.1097/WNF.0b013e318225140c

[12] Committee NIIHSGW, Wall M, McDermott MP, Kieburtz KD, Corbett JJ, Feldon SE et al (2014) Effect of Acetazolamide on visual function in patients with idiopathic intracranial hypertension and mild visual loss: the idiopathic intracranial hypertension treatment trial. JAMA 311(16):1641–1651 24756514 10.1001/jama.2014.3312 PMC4362615

[13] Vaclavu L, Meynart BN, Mutsaerts H, Petersen ET, Majoie C, VanBavel ET et al (2019) Hemodynamic provocation with Acetazolamide shows impaired cerebrovascular reserve in adults with sickle cell disease. Haematologica 104(4):690–699 30523051 10.3324/haematol.2018.206094 PMC6442969

[14] Eskandari D, Zou D, Grote L, Schneider H, Penzel T, Hedner J (2017) Independent associations between arterial bicarbonate, apnea severity and hypertension in obstructive sleep apnea. Respir Res 18(1):130 28659192 10.1186/s12931-017-0607-9 PMC5490198

[15] Zou D, Grote L, Basoglu OK, Verbraecken J, Schiza S, Sliwinski P et al (2023) Arterial bicarbonate is associated with hypoxic burden and uncontrolled hypertension in obstructive sleep apnea - The ESADA cohort. Sleep Med 102:39–45 36599194 10.1016/j.sleep.2022.11.041

[16] Bradford MM (1976) A rapid and sensitive method for the quantitation of microgram quantities of protein utilizing the principle of protein-dye binding. Anal Biochem 72:248–254 942051 10.1016/0003-2697(76)90527-3

[17] Kielkopf CL, Bauer W, Urbatsch IL (2020) Bradford assay for determining protein concentration. Cold Spring Harb Protoc 2020(4):102269 32238597 10.1101/pdb.prot102269

[18] Berry RB, Brooks R, Gamaldo CE, Harding SM, Marcus CL, Vaughn BV (2017) The AASM manual for the scoring of sleep and associated events: rules, terminology and technical specifications. Version 2.4 Darien. American Academy of Sleep Medicine, Illinois

[19] Parkkila S, Parkkila AK, Rajaniemi H, Shah GN, Grubb JH, Waheed A et al (2001) Expression of membrane-associated carbonic anhydrase XIV on neurons and axons in mouse and human brain. Proc Natl Acad Sci U S A 98(4):1918–1923 11172051 10.1073/pnas.98.4.1918 PMC29357

[20] Supuran CT (2008) Diuretics: from classical carbonic anhydrase inhibitors to novel applications of the sulfonamides. Curr Pharm Des 14(7):641–648 18336309 10.2174/138161208783877947

[21] Eskandari D, Zou D, Karimi M, Stenlof K, Grote L, Hedner J (2014) Zonisamide reduces obstructive sleep apnoea: a randomised placebo-controlled study. Eur Respir J 44(1):140–149 24627538 10.1183/09031936.00158413

[22] Gao D, Wang Y, Zhang R, Zhang Y (2021) Efficacy of Acetazolamide for the prophylaxis of acute mountain sickness: A systematic review, Meta-Analysis and trial sequential analysis of randomized clinical trials. Am J Med Sci 361(5):635–645 33587912 10.1016/j.amjms.2020.12.022

[23] Stenkilsson Hoff E (2023) Pharmacological therapy in obstructive sleep apnea: Methodology and interventional aspects of carbonic anhydrase modulation [Doctoral thesis]. Gothenburg: University of Gothenburg

[24] Turnbull CD, Stradling JR (2023) Endotyping, phenotyping and personalised therapy in obstructive sleep apnoea: are we there yet? Thorax 78(7):726–732 37217289 10.1136/thorax-2023-220037

[25] Finnsson E, Arnardóttir E, Cheng W-J, Alex RM, Sigmarsdóttir ÞB, Helgason S et al (2023) Sleep apnea endotypes: from the physiological laboratory to scalable polysomnographic measures. Front Sleep.;2 10.3389/frsle.2023.1188052 PMC12713878 41426478

[26] McNicholas WT, Korkalainen H (2023) Translation of obstructive sleep apnea pathophysiology and phenotypes to personalized treatment: a narrative review. Front Neurol 14:1239016 37693751 10.3389/fneur.2023.1239016 PMC10483231

[27] Geckil AA, Kiran TR, Berber NK, Otlu O, Erdem M, In E (2022) Carbonic anhydrase IX as a marker of disease severity in obstructive sleep apnea. Med (Kaunas).;58(11) 10.3390/medicina58111643 PMC9695925 36422182

[28] Hedner J, Zou D (2022) Turning over a new Leaf-Pharmacologic therapy in obstructive sleep apnea. Sleep Med Clin 17(3):453–469 36150807 10.1016/j.jsmc.2022.06.010

