# Supplementary material for: Reduction of carbonic anhydrase activity is associated with amelioration of obstructive sleep apnea
Source: Sleep Breath. 2025 Sep 4;29(5):278. doi: 10.1007/s11325-025-03430-z (PMC12411314; doi:10.1007/s11325-025-03430-z)
Supplement: Supplementary file 1 — Supplementary Material 1 [file 11325_2025_3430_MOESM1_ESM.docx]

**Reduction of carbonic anhydrase activity is associated with amelioration of obstructive sleep apnea**

Ali M. Komai^1^*, Saliha Musovic^1^*, Kaj Stenlöf^1,2^, Ludger Grote^1,3^, Ding Zou^1^, Jan Hedner^1,3^

^1^Center for Sleep and Wake Disorders, Sahlgrenska Academy, Gothenburg University, Gothenburg, Sweden

^2^Department Internal Medicine, Carlanderska Hospital, Gothenburg, Sweden

^3^Sleep Disorders Center, Respiratory Medicine, Sahlgrenska University Hospital, Gothenburg, Sweden

*=shared first authorship

Running title: Carbonic anhydrase in sleep apnea

Correspondence:

Jan Hedner MD, PhD, FERS

Center for Sleep and Vigilance Disorders

Institute of Medicine, University of Gothenburg

Medicinaregatan 8B, Box 421

SE-40530 Gothenburg

Sweden

Phone: +46 735002077

Email: jan.hedner@lungall.gu.se

**Methods**

**Carbonic anhydrase (CA) activity assessment using a commercially available assay kit.**

In detail, the CA activity kinetic assay kit is used to determine CA activity in biological samples such as hemolysates or cerebrospinal fluid. The assay employs the esterase activity of the CA enzyme on an ester substrate, nitrophenol, which results in release of a chromophore that can be spectrophotometrically quantified at 405 nm. In the current work, a commercially available kinetic assay was evaluated and adapted for assessment of the CA enzyme activity in blood samples. Nitrophenol concentration was monitored for 60 minutes. The advantage of the kinetic assay approach, compared to a standard end-point analysis assay, is the possibility to quantify the enzymatic activity over time with repeated measurements. The specific inhibitor acetazolamide was used as a negative control (provided in the kit) to quantify the specific CA activity. All measurements were performed in 96 well half-area microtiter plates with technical duplicates and read at 405 nm in an end-point mode using the SpectraMax i3x plate reader.
